# Demographic and Component Allee Effects in Southern Lake Superior Gray Wolves

**DOI:** 10.1371/journal.pone.0150535

**Published:** 2016-03-01

**Authors:** Jennifer L. Stenglein, Timothy R. Van Deelen

**Affiliations:** Department of Forest and Wildlife Ecology, University of Wisconsin–Madison, Madison, Wisconsin, United States of America; Michigan Technological University, UNITED STATES

## Abstract

Recovering populations of carnivores suffering Allee effects risk extinction because positive population growth requires a minimum number of cooperating individuals. Conservationists seldom consider these issues in planning for carnivore recovery because of data limitations, but ignoring Allee effects could lead to overly optimistic predictions for growth and underestimates of extinction risk. We used Bayesian splines to document a demographic Allee effect in the time series of gray wolf (*Canis lupus*) population counts (1980–2011) in the southern Lake Superior region (SLS, Wisconsin and the upper peninsula of Michigan, USA) in each of four measures of population growth. We estimated that the population crossed the Allee threshold at roughly 20 wolves in four to five packs. Maximum per-capita population growth occurred in the mid-1990s when there were approximately 135 wolves in the SLS population. To infer mechanisms behind the demographic Allee effect, we evaluated a potential component Allee effect using an individual-based spatially explicit model for gray wolves in the SLS region. Our simulations varied the perception neighborhoods for mate-finding and the mean dispersal distances of wolves. Simulation of wolves with long-distance dispersals and reduced perception neighborhoods were most likely to go extinct or experience Allee effects. These phenomena likely restricted population growth in early years of SLS wolf population recovery.

## Introduction

Allee effects threaten small populations with extinction when growth rate (demographic Allee effect) or a component of individual fitness (component Allee effect) is related positively to population size or density [1, 2]. Demonstrating an Allee effect contradicts expectations that resource abundance is the primary determinant of population growth across all population sizes or densities. A demographic Allee effect is a hump-shaped form of density dependence wherein growth at low relative density shows positive density dependence before transitioning to negative density dependence at a higher relative density [3]. Strong demographic Allee effects exhibit negative population growth at the lowest population sizes whereas weak demographic Allee effects have a pattern of reduced population growth rates (but still positive) at low population sizes. The consequences of strong demographic Allee effects are more severe than weak Allee effects because negative population growth can lead to extinction directly rather than contributing to small-population stochastic risks through slower than expected population growth (a weak Allee effect). A component Allee effect occurs when a component of growth (e.g., survival, reproduction) shows similar positive density dependence at low relative density [3]. Observing a demographic Allee effect indicates the presence of at least one component Allee effect although the reverse may not be true because of compensatory interactions between components of growth [2, 4].

Allee effects are a small population phenomenon and therefore may be particularly influential in reintroduced, newly established, or struggling carnivore populations because carnivores typically exist at low densities, have elaborate social structures, and are sensitive to human activities [5–10]. In addition, small populations may be especially vulnerable to stochastic variation in intrinsic (e.g., age structure) and extrinsic (e.g., habitat) variation. Small populations of carnivores that exhibit long periods of negative or slow growth followed by a sudden increase in growth may indicate the presence of an Allee effect, although frequently it is unidentified or confounded by other sources of variation. Examples of Allee effects identified in small populations of carnivores include: African wild dogs (*Lycaon pictus*) [6], island foxes (*Urocyon littoralis*) [5] and gray wolves in Scandinavia [9] and Yellowstone National Park, USA [7].

Given difficulty in detecting demographic Allee effects in wildlife populations, research has focused on mechanisms influencing component Allee effects. The best evidence for an Allee effect is identification of both demographic effects and component mechanisms, but these cases are rare [11]. In a meta-analysis of 20 studies of Allee effects in mammal populations, five studies could not confirm Allee effects, six examined both demographic and component Allee effects, one study examined only demographic Allee effects, and eight studies examined only component Allee effects [11]. Consequences of Allee effects are reduced population growth, elevated extinction risk, and potential bias in estimation of population parameters; consequently identifying populations prone to Allee effects can improve wildlife conservation efforts [10, 12]. Knowledge of demographic Allee effects helps predict critical numeric population thresholds and elevated extinction risk at low relative density, and knowledge of component Allee effects assists in understanding and potentially mitigating Allee effects.

Reduced breeding interactions at low density is the most commonly cited component Allee effect and usually manifests as a shortage of receptive mate encounters at low-density [13, 14]. Finding a mate is an outcome of individual-based behaviors and decisions on the landscape, and an individual’s perception neighborhood (the range over which an individual can find a mate) is one component of mate-finding [13]. Consequently, individual-based modeling is useful for studying mate-finding and other mechanisms driving Allee effects [13, 15–17].

### Southern Lake Superior (SLS) wolf population

**Fig 1:**
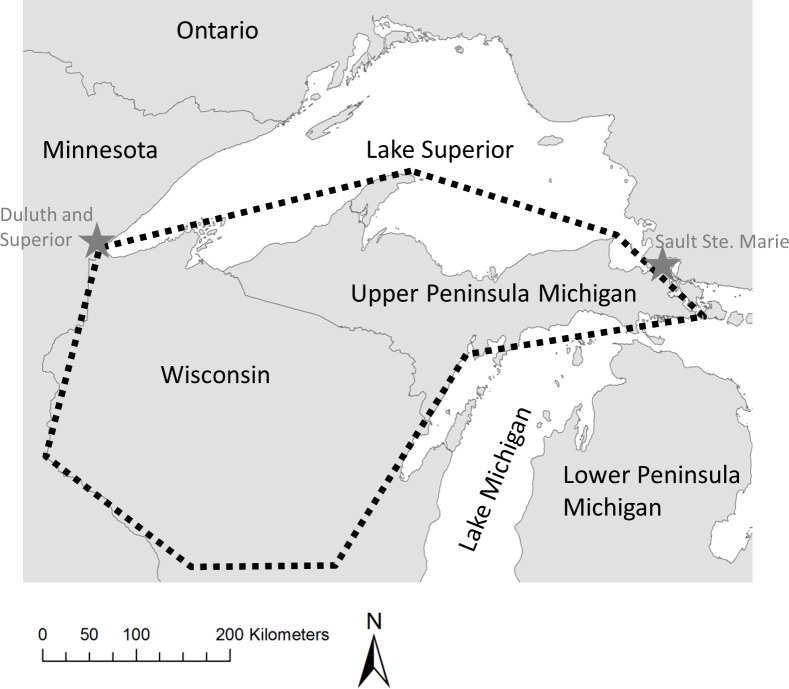
Map of the location of the southern Lake Superior wolf population. Black dotted polygon outlines the primary southern Lake Superior wolf range made up of Wisconsin and the upper peninsula of Michigan (each with currently around 600–800 wolves) and stars show the major cities limiting connectivity to Minnesota and Ontario [26, 27].

We studied the southern Lake Superior (SLS) wolf population (Northern Wisconsin and the upper peninsula of Michigan, USA), which is part of the larger western Great Lakes population of wolves. The SLS region is dominated by mixed forest and has moderate to high quality wolf habitat [18, 19]. SLS wolves are mostly isolated from wolf populations in Minnesota and Ontario because narrow corridors that connect wolf habitat are surrounded by agriculture or water (Lakes Superior and Michigan) and are interrupted by human development (Superior and Duluth in Wisconsin and Minnesota, Sault St. Marie in Michigan and Ontario; Fig 1) [18, 19]. Even so, immigrants are periodically exchanged into the larger population (especially Minnesota) and the SLS population established through natural recolonization from Minnesota into Wisconsin and then to Michigan [20–22]. Recolonization began in the mid-1970s and by 1979 five wolf packs were detected in the northwestern portion of Wisconsin [22]. By the mid-1980s wolves had recolonized the upper peninsula of Michigan, and by the mid-1990s wolves recolonized the central forest region of Wisconsin [23]. Prior to this, the wolf population grew very little and even decreased in some years [24]. Since the mid-1990s, the SLS population has grown at a median rate of 14% per year to >1500 wolves in 2011. An extended period of little or no growth during early recovery is inconsistent with simple negative density dependence across all densities and may suggest an Allee effect. We hypothesized that the interplay between dispersal and mate-finding abilities of wolves varied with density and may have produced an Allee effect similar to that observed in recolonizing wolves in Yellowstone National Park, USA [7]. At low population sizes, high dispersal rates and distances may impede a recovering population if mate-finding is restricted because of a limited ability to detect sparsely distributed mates at a distance, and this combination may lead to an Allee effect [17, 25]. Conversely, a high rate of dispersal matched with increased ability to detect mates rescues a population from an Allee effect and promotes recovery [17].

We were interested in a particular biological hypothesis about wolves’ perception neighborhood in a human-dominated landscape because this is a poorly understood component of wolf behavior. Our objectives were to test for a demographic Allee effect in the early recovery of SLS wolves and, if found, to test whether the high dispersal of colonizing wolves exacerbated a mate-finding Allee effect when the population size was low and dispersers were sparsely distributed. We hypothesized that dispersing wolves had difficulty locating mates during recolonization because potential mates were located mostly outside of the disperser’s perception neighborhood at low density.

## Materials and Methods

### Demographic Allee effect

We tested for a demographic Allee effect in four measures of population growth in SLS wolves (1980–2011) using published data [22, 28, 29]: 1) SLS wolf population size (Michigan and Wisconsin together), 2) Wisconsin wolf population size, 3) number of wolf packs in Wisconsin, and 4) amount of occupied territory in Wisconsin (S1 Appendix). These measures are all highly correlated (i.e., population size, number of packs, and occupied territory all increased over time), although growth rates calculated from these time series are not necessarily highly correlated (S1 Appendix). Therefore, these four measures may reveal different patterns of density dependence that could influence our ability to detect a demographic Allee effect.

For each measure of population growth *i*, for *i* = 1,2,3,4, we fit the relationship between per capita population growth rate, *pgr*_*i*,*t*_ = ln(*N*_*i*,*t*_/*N*_*i*,*t*−1_), and log population size, ln(*N*_*i*,*t*_), in year *t* for *t* = 1981,1982,…,2011, with a penalized spline using Bayesian methods (S2 Appendix) [30] where:

$$pgr_{i,t}∼Normal\left(\mu_{i,t},\sigma_{i}^{2}\right)$$

$$\mu_{i,t}=\beta_{i}\times \text{ln}\left(N_{i,t}\right)+\;\alpha_{i,t,k}\times Z_{i,t,k}$$

The spline portion is *α*_*i*,*t*,*k*_ × *Z*_*i*,*t*,*k*_, where *k* is the number of knots for *k* = 1,2,…,20, and we assigned vague priors $\alpha_{i,t,k}∼Normal(0,\nu_{i}^{2})$ and *ν*_*i*_ ∼ *Uniform*(0,100). Also, we assigned vague priors *β*_*i*_ ∼ *Normal*(0,100^2^) and *σ*_*i*_ ∼ *Uniform*(0,100). Heuristically, using a spline enables the data to determine the shape of the relationship between *pgr*_*i*,*t*_ and ln(*N*_*i*,*t*_) instead of assuming a functional form for this relationship *a-priori* through a parametric (e.g., linear, quadratic) model. Evidence for an Allee effect would be a hump-shaped spline [25]. A spline crossing the x-axis at two non-negative values would identify the Allee threshold (low-density unstable equilibrium also called the extinction threshold) and the carrying capacity (high-density stable equilibrium), respectively [3]. We chose to use penalized splines, specifically low-rank thin-plate splines in our analysis because of their good mixing properties in the Markov Chain Monte Carlo (MCMC) chains of a Bayesian analysis [30, 31].

We ran the models in program R (version 2.14) [32], library ‘rjags’ [33] with program JAGS (version 3.3.0) [34]. We ran three MCMC chains for each model for 150,000 iterations and discarded the first 100,000 iterations as burn-in (the testing period that is thrown out prior to stabilization of chains). For each model, we assessed convergence using visual inspection of chain mixing and univariate ($\hat{R}$) and multiple potential scale reduction factors (${\hat{R}}^{p}$, where *p* is the number of parameters) [35, 36]. Generally, convergence is adequate when upper 97.5% confidence limits of the $\hat{R}$s and ${\hat{R}}^{p}$ statistics are close to 1 and here we declared convergence attained if the upper 97.5% confidence limits of all $\hat{R}$s and ${\hat{R}}^{p}$ were <1.1 [36].

### Component Allee effect

**Fig 2:**
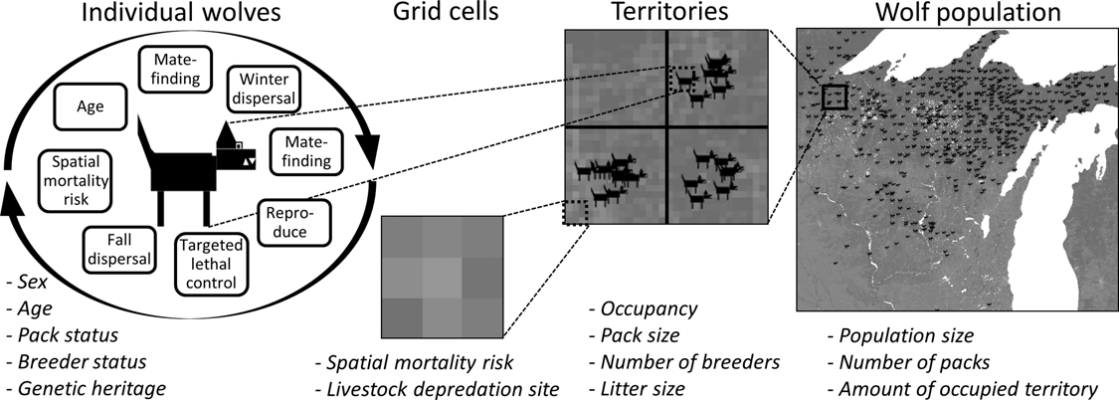
Depiction of an individual-based spatially explicit model for growth of the southern Lake Superior wolf population [37]. The hierarchical levels of organization are the individual wolves, grid cells that make up the landscape, territories, and wolf population and the lists (e.g., sex, age, pack status) are the variables that characterize each level.

We evaluated hypothetical mechanisms [7] leading to an Allee effect by simulating population growth under various mate-finding distances and dispersal distance functions for wolves in an individual-based spatially explicit (IBSE) model of the SLS wolf population [37]. We derived parameters for the model from empirical research specific to the Great Lakes wolf population [22] using NetLogo (version 4.1) [38]. Our model is described in detail in Stenglein, Gilbert (37) following the Overview, Design, and Details protocol (ODD) [39, 40]. Here, we give a brief overview (Table 1; Fig 2).

**Table 1:** Life history events and sequence of events for simulated southern Lake Superior wolves in an individual-based spatially explicit model [37].

Life history event	Sequence	Description
Mate-finding	1, 3	A breeding wolf in a territory^a^, if not mated (mate died in previous year), searches for an unrelated wolf of the opposite sex, first in their own territory and then within their perception neighborhood of up to 1, 2, 3, 4 or 5 territories away, depending on the simulation. Next, any non-breeding wolves that are unrelated to other wolves within its territory or that are located outside of breeding range^b^ will look for each other within their perception neighborhood, pair up if unrelated and of the opposite sex, move to the nearest territory, and establish themselves as the breeding pair if there are no other breeders in that territory.
Winter dispersal	2	All wolves that are not breeders and without other wolves nearby disperse to increase their chances of finding a mate by choosing a random direction and moving a distance drawn from lognormal distribution with the log mean equal to 3.23, 3.92, or 4.61 depending on the simulation and log standard deviation equal to 1.01.
Reproduce	4	All breeding females reproduce a number of pups drawn from a normal distribution with mean equal to 5.41 and standard deviation equal to 0.79 and rounded to the nearest whole number. The sex of each pup is chosen randomly.
Targeted lethal control	5	To simulate the lethal control of wolves to alleviate livestock depredation in the summer months, wolves are killed from within 5 km of areas where there have been reported livestock depredations in Wisconsin in the late 2000s. A total of 10% of the last winter count of wolves in Wisconsin are killed from these high depredation areas once the simulated population reaches 350 wolves in Wisconsin.
Fall dispersal	6	To simulate resource limitation, the number of non-breeding wolves within a pack in excess of 10 wolves will disperse out of their natal pack by choosing a random direction and moving a distance drawn from a lognormal distribution with log mean equal to 3.23, 3.92, or 4.61 depending on the simulation and log standard deviation equal to 1.01. If these wolves do not disperse far enough to leave the pack, they die.
Spatial mortality risk	7	Wolves survive with a probability prescribed by the spatial mortality risk determined by local road density and amount of agriculture [37].
Age	8	Wolves age each year and die if they reach 12 years of age.

^*a*^ 225 km^2^ that support up to 1 pack and exist in areas with low background risk. There are 363 potential territories with 151 of them in Wisconsin.

^*b*^ Areas of Minnesota, Wisconsin, and the upper peninsula of Michigan where the spatial mortality risk is <0.75.

In our IBSE model, unmated individual male and female adult wolves experienced an annual cycle of life history events culminating in a goal of finding a mate and establishing a territory to become a reproducing pack (Table 1). Simulated unmated wolves could move around the model landscape during mate-finding, winter dispersal, and fall dispersal events. Wolves could die from targeted lethal control efforts, fall dispersal mortality, spatially varying mortality risk reflecting human activity, and aging (Table 1). Wolves could enter the simulation annually through reproduction and through a winter dispersal event by replacing a number of immigrants equal to the number of emigrants that dispersed beyond the bounds of the simulated landscape (Table 1; Fig 2). Each repetition began with 20 breeding pairs in territories in Minnesota and ran for 40 years or until all wolves died. We varied the individual-level perception neighborhoods (5 categories) and dispersal distances (drawn from a lognormal distribution with varying log mean parameters (3 categories; Table 1) and ran 100 repetitions for each of the 3 × 5 = 15 simulations for a total of 1500 repetitions.

**Fig 3:**
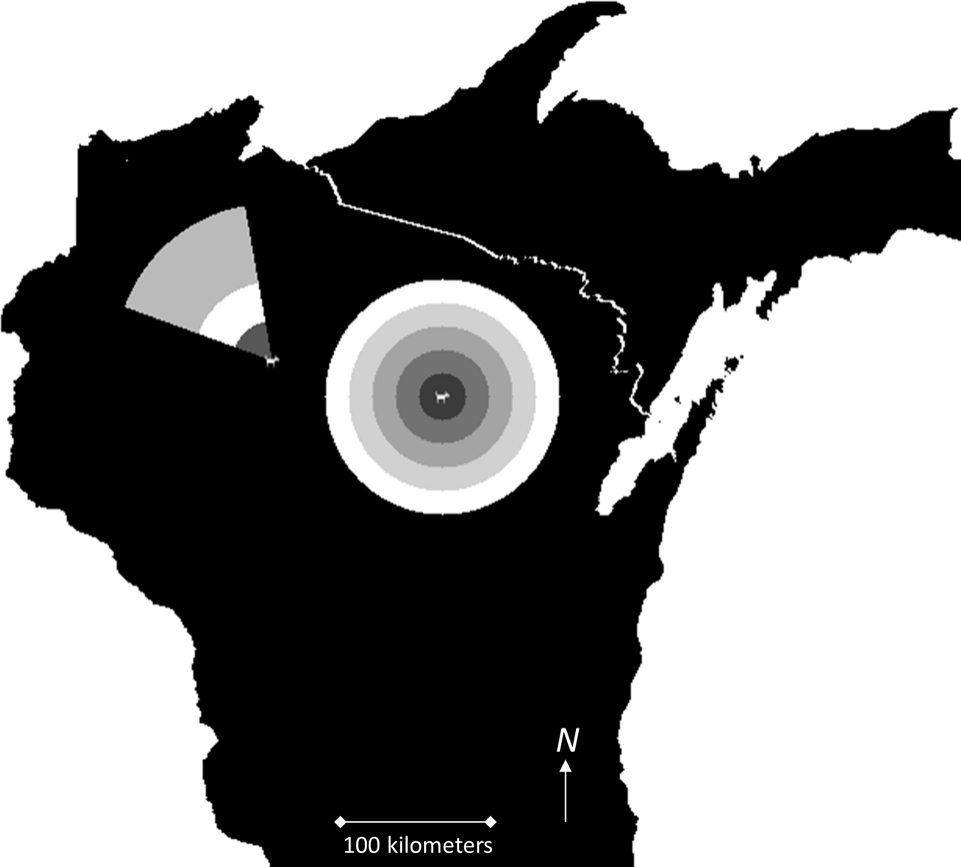
Simulations for an individual-based spatially explicit model for southern Lake Superior wolves. We varied perception neighborhoods where simulated wolves could search for mates 1, 2, 3, 4 and 5 territories away (concentric circles) and the log mean parameter in the lognormal distribution used to calculate individual dispersal distance with average dispersal distances of 25, 50, and 100 kilometers (sectors) on a simulated landscape.

A wolf’s perception neighborhood for detecting mates is largely unknown. We varied this parameter in the IBSE model in territory-based increments from a perception neighborhood of one territory away (15 km) to five territories away (75 km; Fig 3). Dispersal distance is better understood for Great Lakes wolves, but there are still many uncertainties with defining a dispersal event and determining how to deal with bias associated with radio-collared wolves that may have dispersed but are lost from radio-contact. We fit a lognormal distribution to 110 observations of Great Lakes wolf dispersal distances [20] and used the maximum likelihood estimates for the mean, *d*_*ave*_, and standard deviation, *s*: ln(*d*_*ave*_) = 3.92 and ln(*s*) = 1.01. We took *d*_*ave*_ to be our best estimate of true mean dispersal distance, and then considered alternate dispersal distance functions where mean dispersal distance was half of *d*_*ave*_, *d*_*low*_, and where dispersal distance was double *d*_*ave*_, *d*_*high*_ (Fig 3).

To test whether mate-finding limitations would lead to a demographic Allee effect, we looked for evidence of Allee effects in the relationship between simulated per capita population growth and the SLS population size. For each repetition, we calculated *pgr*_*t*_ and plotted *pgr*_*t*_ versus ln(*N*_*t*_) for *t* = 2,3,…,*T* where *T* was the number of years in the time series and *N*_2_ was the population size in the first year the simulated SLS population was ≥15 wolves because this was the minimum number of wolves detected in the SLS population since wolf recovery in the SLS region [22]. For each plot, we fit a cubic smoothing spline with six knots using the function “smooth.spline” with its default values in program R. We categorized each simulation outcome as: 1) ‘extinct’ when we could not assess because simulations never reached 15 wolves or ≥10 data points, 2) ‘strong Allee effect’ when the spline started with a positive slope and negative values for per capita growth, 3) ‘weak Allee effect’ when the spline started with a positive slope and positive values for per capita growth, or 4) ‘no evidence for an Allee effect’ when the spline started with a negative slope.

We explored how the probability of evidence for an Allee effect, *p*_*i*_ where *i* = 1,2,…1500 indexed the repetition, was affected by the choice of perception neighborhood and mean dispersal distance with a logistic regression model:

$$Y_{i}∼Bernoulli(p_{i})$$

$$logit(p_{i})=\;\beta_{0}+\;x_{1}\times \beta_{1}+\;x_{2}\times \beta_{2}+\cdots +\;x_{K}\times \beta_{K}$$

The response *Y*_*i*_ = 1 if there was evidence for a strong or weak Allee effect. The predictors *x*_*k*_ for *k* = 1,2,…,*K* for *K* total predictors were dummy variables for the different perception neighborhood and dispersal distance combinations. We considered aggregating some categories depending on whether there appeared to be an interaction between perception neighborhood and dispersal distance. We assigned vague priors to the parameters, *β*_*k*_ ∼ *normal*(0,100^2^). We ran this model in a Bayesian framework following the methods outlined above.

## Results

### Demographic Allee effect

**Fig 4:**
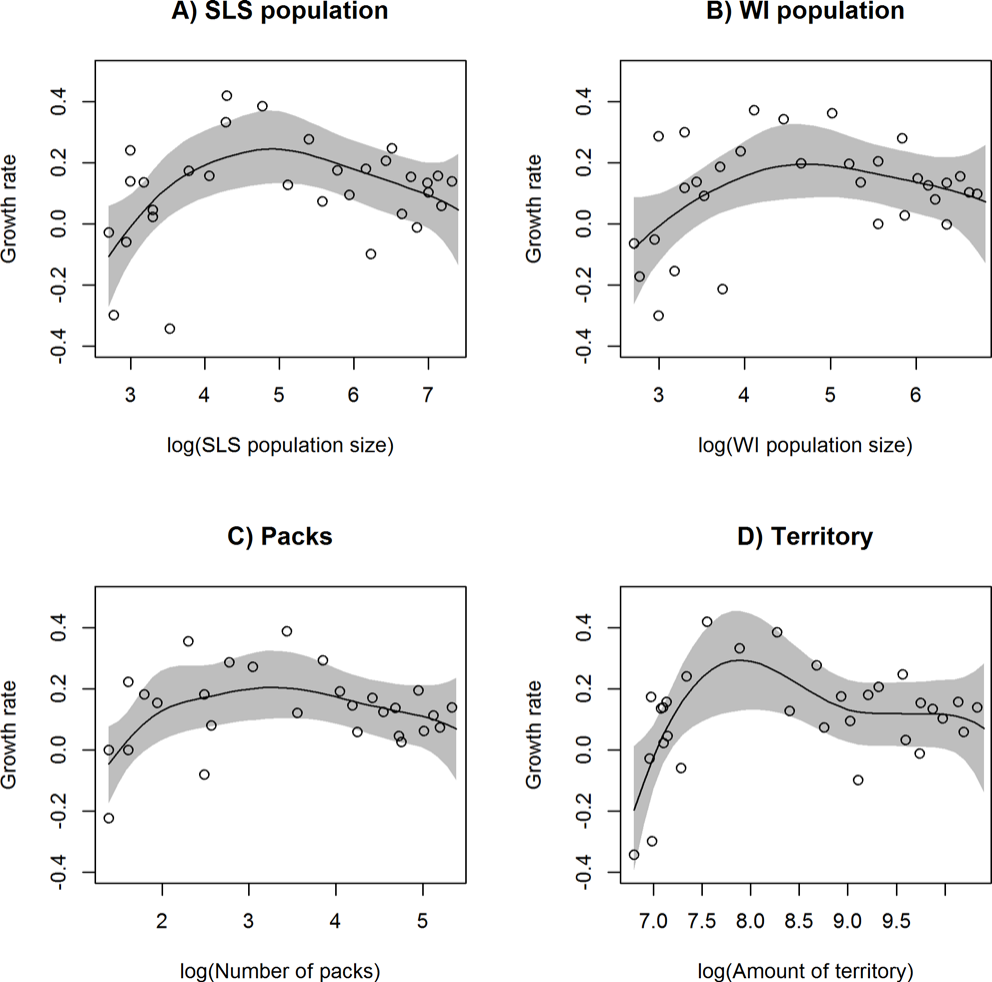
Splines fit to growth versus population size of the southern Lake Superior wolf population in 1980–2011. Fitted curves with 95% credible intervals from splines fit to the relationship between per capita growth and four measure of population size for gray wolves in the southern Lake Superior wolf (SLS) population (A) and Wisconsin (B), including the number of packs (C) and the proportion of occupied territory in Wisconsin (D).

The MCMC algorithms converged adequately for all models (upper 97.5% estimates of $\hat{R}$ were < 1.04 for all parameters, and the overall ${\hat{R}}^{p}$ statistics were < 1.02 for all models). Strong demographic Allee effects were evident from all models because the splines fit to the data were all hump-shaped and the spline changed from negative to positive growth rates at a low population size (i.e., positive Allee thresholds; Fig 4). No models had fitted splines that passed into negative growth rates at high population size which would have provided an estimate of carrying capacity (Fig 4). Mean posterior fitted values from the SLS population dataset and the Wisconsin population dataset both had an Allee threshold around 1987–1988 when there were approximately 20 wolves in these populations. Mean posterior fitted values reached maximum growth in the SLS population in 1994–1995 with approximately 135 wolves and in 1996–1997 in the Wisconsin population with approximately 111 wolves. For the pack dataset, the Allee threshold was estimated to have occurred slightly earlier in 1985–1986 when there were four to five packs of wolves, and maximum growth was estimated to have occurred in 1995–1996 when there were 26–27 packs of wolves in Wisconsin. The territory dataset had the latest estimated Allee threshold in 1990–1991 when there was approximately 1100 km^2^ of occupied wolf territory in Wisconsin and the maximum growth was reached in 1993–1994 when there was approximately 2705 km^2^ of occupied wolf territory.

### Component Allee effect

Of the 1500 repetitions from the IBSE model, 33 (2.2%) of them went functionally extinct in the sense that they could not be assessed because the population did not grow to ≥15 wolves or did not persist for ≥10 years with ≥15 wolves. All extinctions occurred when the perception neighborhood was simulated to be one territory away, and extinction was >4 times as frequent in the simulations with high dispersal distance compared to average or low dispersal distances (Table 2). There were 545 (36.3%) repetitions with a probable Allee effect and there were approximately twice as many weak Allee effects compared to strong Allee effects. A third of the strong Allee effects occurred under high dispersal when the perception neighborhood was one territory away. Of the strong Allee effects, 72.1% occurred when the perception neighborhood was one territory away and 48.1% of them occurred under high dispersal distance (Table 2). There was little difference in the number of probable Allee effects for simulations that had perception neighborhoods for ≥3 territories away (Table 2).

**Table 2:** Number of repetitions with evidence for Allee effects from simulations of an individual-based spatially explicit model for gray wolves [37] in the southern Lake Superior region.

			Allee effect		
Perception neighborhood (territories)	Mean dispersal distance	Extinct	Strong	Weak	No evidence
1	Low	1	31	24	44
1	Ave	6	38	17	39
1	High	26	63	3	8
2	Low	0	8	26	66
2	Ave	0	4	21	75
2	High	0	15	35	50
3	Low	0	3	32	65
3	Ave	0	7	25	68
3	High	0	7	27	66
4	Low	0	2	19	79
4	Ave	0	1	28	71
4	High	0	2	24	74
5	Low	0	1	28	71
5	Ave	0	0	30	70
5	High	0	1	23	76

The perception neighborhood for mates was varied from 1–5 territories away and the mean dispersal distance from the dispersal function was either low (25 km), average (50 km), or high (100 km). Please see text for category descriptions.

**Fig 5:**
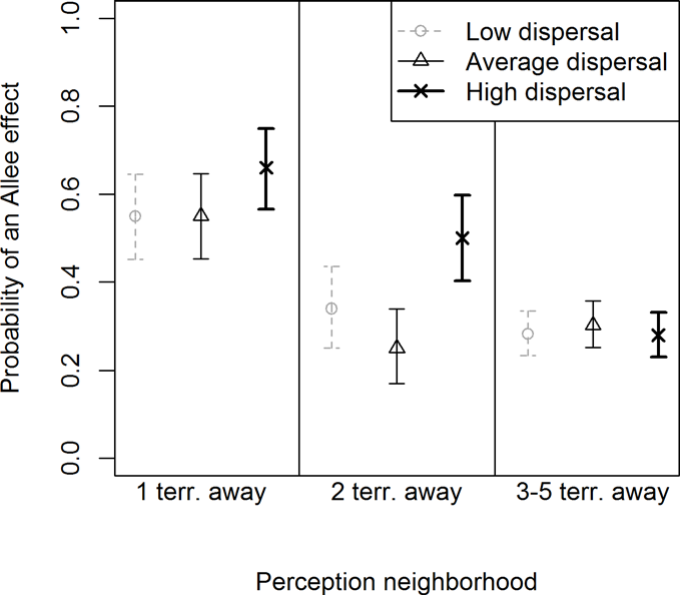
The probability of an Allee effect from simulations of an individual-based spatially explicit model. Posterior mean and 95% credible intervals of the probability of an Allee effect from simulations varying the perception neighborhood for mate-finding as 1, 2 or >3 territories (terr) away and the mean dispersal distances as low (25 km), average (50 km) and high (100 km) in an individual-based spatially explicit model for gray wolves in the southern Lake Superior region [37].

Consequently in the logistic regression, we grouped the simulations with perception neighborhoods ≥3 territories away. The MCMC algorithm converged adequately, and the upper 97.5% estimates of $\hat{R}$ and ${\hat{R}}^{p}$ were 1. Simulations with perception neighborhoods of ≥3 territories away for low, average or high dispersal distances and simulations with a perception neighborhood of two territories away for low and average dispersal distances were least likely to show evidence of an Allee effect (Fig 5). High dispersal distance simulations with a perception neighborhood of two territories away were just as likely to have an Allee effect as to have no evidence for an Allee effect. Simulations with perception neighborhoods of one territory away and low and average dispersal distances were more likely to have an Allee effect than not (Fig 5). Finally, the simulation most likely to go extinct with a perception neighborhood of one territory away and high dispersal was also the simulation with the highest probability of Allee effects (Fig 5).

## Discussion

We detected a strong demographic Allee effect in the SLS wolf population. Simulations from an IBSE model suggested that the Allee effect could have resulted from wolves dispersing far from population centers and into vacant territories leading to an inability to find mates [7]. In addition, simulation scenarios that produced Allee effects associated with increased simulation failures (extinctions of simulated populations). The combination of high dispersal potential and a restricted perception neighborhood for mate-finding may have restricted population growth in early years of population recovery in SLS wolves. Social carnivores can be particularly vulnerable to Allee effects because of their need for conspecifics in hunting and rearing of young and because they often exist at low densities [5, 8]. Hence, carnivore recovery likely requires careful consideration of Allee effects because of the numerous, intertwined factors that influence dynamics of the population related to population size and density [8, 12, 25].

Demographic Allee effects are notoriously difficult to detect because of the need for a long-term dataset of population counts spanning a range of densities and the potential complications of observer error and demographic stochasticity which can be prevalent at low population sizes [14, 25, 41, 42]. Therefore, it is especially notable that we detected a demographic Allee effect in the SLS wolf population. Further, we detected a strong demographic Allee effect which allowed us to estimate the Allee threshold in this population. From the relationship between per capita growth and population size in the wolf population, we estimated an Allee threshold was passed in the mid- to late-1980s, nearly a decade into population recovery. Hence, the SLS wolf population was probably at or below the Allee threshold for the first decade of reestablishment and could have just as likely become extinct as successfully recolonized during this time. It may be that immigration from Minnesota or Ontario prevented extinction by supplementing population growth sufficiently to exceed the Allee threshold [20]. The population achieved maximum growth and switched from positive to negative density dependence in the mid-1990s coincident with colonization of the central forest region of Wisconsin and the upper peninsula of Michigan–the last remaining patches of high quality habitat [18, 23] and may support an interpretation that growth at high relative density was limited by the availability of high quality habitat or vacant territories.

Examples of mate-finding Allee effects leading to a demographic Allee effect for a species that has evolved life-history strategies to improve mate-finding probability are rare [14]. We found compelling evidence of wolves, a territorial and vagile species with long distance communication capabilities that plausibly facilitate mate-finding (howling, scent-marking), experiencing mate-finding and demographic Allee effects. Wolves’ ability to detect mates through communication and searching was probably evolved because until recently wolves were the most widely-distributed terrestrial species without many limitations on their movement or establishment [43]. Presently, wolf populations are reduced to fragments of their historic range. In the SLS region, the probability of mortality from human activity has restricted population expansion into a corridor of the northern forested portion of Wisconsin and the upper peninsula of Michigan where human influence is relatively reduced (small portion of historic range) [22]. Hence anthropogenic factors have excluded wolves from a region where population growth would not otherwise be so limited. In a human-dominated landscape, wolf use of space interacts with spatially varying risk of human-caused mortality [44] such that unnatural sparseness or low density likely inhibits mate-finding capacity.

Early reestablishment of the SLS wolf population probably was not slowed because of lack of territory (1547 wolves in the SLS region in 2011) or lack of food [24]. We evaluated potential for a mate-finding Allee effect in the recolonizing SLS wolf population because it is the most-cited Allee effect mechanism [13] and other wolf populations have documented or suspected mate-finding Allee effects [7, 9]. Additionally, we assessed changes in fecundity and the proportion of lone wolves over time (Stenglein unpublished) in Wisconsin’s wolf population data [22] and found no evidence of other Allee effect mechanisms. We did not find reduced fecundity in pups per pack or in the proportion of breeding females in the population pre-1995 compared to 1995–2007 (Stenglein unpublished). However, the proportion of lone wolves prior to 1995 (roughly 10% of the population) was higher compared to 1995–2007 when only 4% were lone wolves [22]. The difference in proportion of lone wolves could be due to sampling and detection issues; however a real difference provides support for a mate-finding component Allee effect in early recovery because it suggests that wolves had difficulty finding mates at low densities, resulting in more lone wolves.

Pathogens with long infection cycles or stable reservoirs can persist in small populations and impede population growth [45]. A population affected by pathogens and Allee effects may be more prone to extinction than a population suffering from Allee effects alone and these effects can be more pronounced in social species, like wolves [8, 46–48]. We did not model pathogens explicitly as a source of mortality for simulated wolves in our individual-based model, although we suggest this as an extension if empirical data on density dependent population effects in social carnivore become available. Wolf pathogens identified in the SLS region include canine parvovirus, canine distemper virus, mange, blastomycosis, Lyme disease, anaplasmosis, canine ehrlichiosis and heartworm [49–51]. During early population recovery in the mid-1980s, canine parvovirus was detected in the Wisconsin wolf population and may have reduced survival of wolf pups [52]. In Minnesota, a negative correlation between number of pups captured and canine parvovirus seroprevalence was found during this same time period, also suggesting a reduction in pup survival [53, 54]. However, no population-level effect was detected during the time of the canine parvovirus outbreak suggesting compensatory interactions with other mortalities [54]. In wild wolf populations, canine parvovirus has trivial impacts on adult survival and population size despite elevated pup mortality [51, 54, 55].

Factors other than Allee effect mechanisms could cause observations of negative population growth followed by a sudden increase in growth. Observer error estimating the four measures of population growth we used could have contributed to the appearance of an Allee effect. When the wolf population was small, it may have been more difficult to count wolves, packs and occupied territory. At small population sizes, failing to count just one pack and then finding and counting it in the next year could lead to the appearance of substantial population growth which would be due to observer error rather real growth. Demographic stochasticity in small populations can result in perceived Allee effects [56]. However, demographic stochasticity itself is sometimes considered an Allee effect mechanism when a skewed sex ratio occurring by chance results in mate limitation and subsequent decreased fitness [57, 58]. Our individual-based model incorporated demographic stochasticities by drawing litter sizes, sex assignments of pups, dispersal distances and survival from characteristic probability distributions which resulted in some repetitions within a simulation showing evidence for an Allee effect and others not. Even so, an overall pattern emerged from the simulations that supported a mate-finding Allee effect.

A potential improvement to our model would be to incorporate a more sophisticated mate-finding process for wolves. We treated mate-finding simply in our individual-based model; individual wolves were able to search for mates up to two times each year but only within a maximum distance of their current location and not during dispersal events. This resulted in a circular search area for mates and was not based on landscape information. Wolves may travel most often in long, linear routes [59]. Similarly wolves and other mammals may move across paths of least resistance or choose paths through preferred habitat [20, 60]. However, to our knowledge, nobody has measured the shape of a wolf’s perception neighborhood. If perception is based on auditory cues (howling) it could be relatively independent of habitat and therefore circular, and especially if howling can be detected by wolves at great distances. As understanding of the mate-finding process for wolves improves, modelers can design and parameterize a more sophisticated mate-finding process that could incorporate measures of landscape resistance to allow simulated wolves to locate mates in a more informed way [60].

The confluence of long-term datasets and computational power that can support individual-based models expanded opportunities for studying and understanding population dynamics. Splines are an improvement over parametric models when looking for evidence for a demographic Allee effect because they provide useful flexibility in letting the data determine functional relationships [25, 30]. Once a demographic Allee effect is detected, hypothesized mechanisms leading to the Allee effect should be evaluated, and individual-based models provide a useful framework for testing these hypotheses. A well-parameterized individual-based model can be used to study specific mechanisms as well as the emergent population properties to which they contribute [44] and can inform important conservation concerns such as long-term population viability, or how novel mortalities that vary in space and time (e.g., hunting, illegal killing, infectious disease) will affect the population [37, 61, 62].

The SLS wolf population size is >60 times higher than the Allee threshold that we detected; therefore it is very unlikely that the SLS wolf population size would be reduced to a level where it would be prone to Allee effects in the near future [26, 29]. However, as wolves become more established in the SLS region, they are moving to other areas, including the lower peninsula of Michigan, southern Wisconsin and surrounding states [22, 26, 63]. If conservation and expansion for wolves is a goal in these areas, conservationist may need to monitor population growth data in newly established populations to infer whether Allee effects are occurring. Understanding whether an Allee threshold exists (and at what population size) will help predict population growth and expansion probabilities in new areas. Further, an IBSE model has potential to test hypotheses about dynamics other than Allee effects in small populations, such as the effect of demographic stochasticity in newly established populations and the effect of inbreeding in small populations [64, 65].

Difficulty in detecting Allee effects does not diminish the importance that they may play in the dynamics of small and recovering populations, and particularly in the case of social carnivores where social facilitation is a key feature of reproduction [6–8, 14]. Rigorous simulation techniques (e.g., Bayesian MCMC approaches, individual-based models) may offer an optimal strategy for integrating field and published data on population dynamics. Our analysis of complexities in the density dependent growth of the SLS wolf population suggests that similar approaches might provide new insights on the dynamics of small and sparse populations.

## Supporting Information

S1 Appendix Datasets of southern Lake Superior wolf population growth. (DOCX)

S2 Appendix Bayesian spline model with R code. (DOCX)

S1 Fig Individual-based model simulation results. Simulation with a perception neighborhood of 1 territory away and an average dispersal distance of 50 km (average dispersal).
(TIFF)

S2 Fig Individual-based model simulation results. Simulation with a perception neighborhood of 1 territory away and an average dispersal distance of 100 km (high dispersal).
(TIFF)

S3 Fig Individual-based model simulation results. Simulation with a perception neighborhood of 1 territory away and an average dispersal distance of 25 km (low dispersal).
(TIFF)

S4 Fig Individual-based model simulation results. Simulation with a perception neighborhood of 2 territories away and an average dispersal distance of 50 km (average dispersal).
(TIFF)

S5 Fig Individual-based model simulation results. Simulation with a perception neighborhood of 2 territories away and an average dispersal distance of 100 km (high dispersal).
(TIFF)

S6 Fig Individual-based model simulation results. Simulation with a perception neighborhood of 2 territories away and an average dispersal distance of 25 km (low dispersal).
(TIFF)

S7 Fig Individual-based model simulation results. Simulation with a perception neighborhood of 3 territories away and an average dispersal distance of 50 km (average dispersal).
(TIFF)

S8 Fig Individual-based model simulation results. Simulation with a perception neighborhood of 3 territories away and an average dispersal distance of 100 km (high dispersal).
(TIFF)

S9 Fig Individual-based model simulation results. Simulation with a perception neighborhood of 3 territories away and an average dispersal distance of 25 km (low dispersal).
(TIFF)

S10 Fig Individual-based model simulation results. Simulation with a perception neighborhood of 4 territories away and an average dispersal distance of 50 km (average dispersal).
(TIFF)

S11 Fig Individual-based model simulation results. Simulation with a perception neighborhood of 4 territories away and an average dispersal distance of 100 km (high dispersal).
(TIFF)

S12 Fig Individual-based model simulation results. Simulation with a perception neighborhood of 4 territories away and an average dispersal distance of 25 km (low dispersal).
(TIFF)

S13 Fig Individual-based model simulation results. Simulation with a perception neighborhood of 5 territories away and an average dispersal distance of 50 km (average dispersal).
(TIFF)

S14 Fig Individual-based model simulation results. Simulation with a perception neighborhood of 5 territories away and an average dispersal distance of 100 km (high dispersal).
(TIFF)

S15 Fig Individual-based model simulation results. Simulation with a perception neighborhood of 5 territories away and an average dispersal distance of 25 km (low dispersal).
(TIFF)

S1 Table Summary individual-based model simulation results. (XLSX)
