# Correction: Demographic and Component Allee Effects in Southern Lake Superior Gray Wolves

**DOI:** 10.1371/journal.pone.0269290

**Published:** 2022-05-26

**Authors:** Jennifer L. Stenglein, Timothy R. Van Deelen

In the fourth paragraph of the discussion, two references to “Stenglein unpublished” are made. The authors provide these data here as Supporting Information file: S3 Appendix. The correct paragraph reads: Early reestablishment of the SLS wolf population probably was not slowed because of lack of territory (1547 wolves in the SLS region in 2011) or lack of food [24]. We evaluated potential for a mate-finding Allee effect in the recolonizing SLS wolf population because it is the most-cited Allee effect mechanism [13] and other wolf populations have documented or suspected mate-finding Allee effects [7, 9]. Additionally, we assessed changes in fecundity and the proportion of lone wolves over time (S3 Appendix) in Wisconsin’s wolf population data [22] and found no evidence of other Allee effect mechanisms. We did not find reduced fecundity in pups per pack or in the proportion of breeding females in the population pre-1995 compared to 1995–2007 (S3 Appendix). However, the proportion of lone wolves prior to 1995 (roughly 10% of the population) was higher compared to 1995–2007 when only 4% were lone wolves [22]. The difference in proportion of lone wolves could be due to sampling and detection issues; however a real difference provides support for a mate-finding component Allee effect in early recovery because it suggests that wolves had difficulty finding mates at low densities, resulting in more lone wolves.

S3 Appendix has been added to the Supporting Information. It can be viewed below.

The authors provide the following additional information:

Four measures of population growth were assessed in [1]. All four data sets were derived from a combination of methods employed by Wisconsin Department of Natural Resources (DNR) since 1979, including snow-track surveys, aerial radiotracking, summer howling surveys, and wolf observations. Details are found in [2] and data are available in S1 Appendix.

The wolf count data that was used in [1], cited and included in its entirety in S1 Appendix, is the official Wisconsin DNR time series of annual wolf population estimates. There are no changes or improvements of any consequence to the Wisconsin wolf counting methodology over time that would have resulted in artificial increases in wolf numbers. This data set is owned and managed by Wisconsin DNR and the authors worked with Wisconsin DNR in publishing [1]. The authors acknowledged Wisconsin DNR in [1] for their contributions to this work.

## Supporting information

S3 Appendix Analysis of changes in fecundity and proportion of lone wolves over time. (DOCX)
