# Supplementary material for: Demographic and Component Allee Effects in Southern Lake Superior Gray Wolves
Source: PLoS One. 2016 Mar 1;11(3):e0150535. doi: 10.1371/journal.pone.0150535 (PMC4801012; doi:10.1371/journal.pone.0150535)
Supplement: S1 Appendix — (DOCX) [file pone.0150535.s001.docx]

**S1 Appendix.** Datasets of SLS wolf population growth.

We describe the 4 datasets that we used in our analysis of growth rate versus size of the southern Lake Superior (SLS) wolf population in 1980 – 2011 (Table S1.1). Our question was whether there was a demographic Allee effect present in population growth data. Evidence for a demographic Allee effect can be found from the graph of growth rate versus size of a population. In the SLS wolf population, we have 4 potential measure of population size: 1) estimated wolf population size in the SLS region (Michigan and Wisconsin; B. Roell, pers. Comm.) [[1-3](#_ENREF_1)], 2) estimated wolf population size in Wisconsin, 3) number of wolf packs in Wisconsin [[1](#_ENREF_1), [3](#_ENREF_3)], and 4) amount of occupied territory in Wisconsin (Table S1.1) [[1](#_ENREF_1)]. Wisconsin Department of Natural Resources uses territory mapping for wolf population monitoring [[1](#_ENREF_1)]. A result of territory mapping is a shapefile of pack territories in Wisconsin. We calculated the amount of occupied territory in Wisconsin each year as the total sum of pack areas mapped in each year.

The datasets of SLS population size, Wisconsin population size, number of packs, and occupied territory were highly correlated with each other ($\rho=0.996-0.999$; Fig. S1.1). However, the growth rates calculated from these datasets were much less correlated (Table S1.2, Fig. S1.2). This lack of strong correlation among some of the measures of the SLS population prompted us to assess all of these measures for evidence of an Allee effect. If all measures were in agreement about whether or not an Allee effect was present, then that would lend strength to our argument of an Allee effect in this population. If not all measures were in agreement, then we would be more skeptical about our findings.

**Table S1.1.** Datasets from the southern Lake Superior (SLS) wolf population, USA (1980 – 2011) used in an analysis of growth rate versus size. Number (No.) of packs and amount of occupied territory data are from Wisconsin (WI).

| Year | SLS pop size | WI pop size | No. of packs | Territory (km^2^) |
| --- | --- | --- | --- | --- |
| 1980 | 28 | 28 | 5 | 1037 |
| 1981 | 24 | 24 | 5 | 1188 |
| 1982 | 27 | 27 | 4 | 1216 |
| 1983 | 20 | 20 | 5 | 1547 |
| 1984 | 19 | 19 | 4 | 1460 |
| 1985 | 16 | 16 | 4 | 1082 |
| 1986 | 15 | 15 | 5 | 1052 |
| 1987 | 20 | 20 | 5 | 1211 |
| 1988 | 27 | 27 | 6 | 1269 |
| 1989 | 34 | 31 | 7 | 900 |
| 1990 | 44 | 34 | 10 | 1072 |
| 1991 | 58 | 41 | 12 | 1255 |
| 1992 | 73 | 52 | 13 | 1910 |
| 1993 | 72 | 42 | 12 | 2666 |
| 1994 | 118 | 61 | 16 | 3923 |
| 1995 | 166 | 86 | 21 | 4459 |
| 1996 | 221 | 105 | 31 | 5891 |
| 1997 | 264 | 151 | 35 | 6342 |
| 1998 | 323 | 184 | 47 | 7561 |
| 1999 | 380 | 211 | 57 | 8313 |
| 2000 | 475 | 259 | 66 | 9958 |
| 2001 | 508 | 259 | 70 | 9023 |
| 2002 | 621 | 343 | 83 | 11096 |
| 2003 | 674 | 353 | 94 | 14216 |
| 2004 | 770 | 410 | 108 | 14683 |
| 2005 | 870 | 465 | 113 | 17122 |
| 2006 | 938 | 504 | 116 | 16915 |
| 2007 | 1086 | 577 | 141 | 19350 |
| 2008 | 1096 | 576 | 150 | 21473 |
| 2009 | 1250 | 673 | 168 | 25139 |
| 2010 | 1304 | 747 | 181 | 26692 |
| 2011 | 1511 | 824 | 208 | 30697 |

**Table S1.2.** Pearson’s correlation ($\rho$) and p-value among the growth rates calculated from the 4 measures of wolf population size in the southern Lake Superior (SLS) region, USA in 1980 – 2011.

| Growth rate 1 | Growth rate 2 | $\rho$ | p-value |
| --- | --- | --- | --- |
| SLS pop growth | WI pop growth | 0.220 | 0.235 |
| SLS pop growth | Pack growth | 0.910 | < 0.001 |
| SLS pop growth | Territory growth | 0.363 | 0.045 |
| WI pop growth | Pack growth | 0.296 | 0.107 |
| WI pop growth | Territory growth | 0.443 | 0.013 |
| Pack growth | Territory growth | 0.301 | 0.100 |


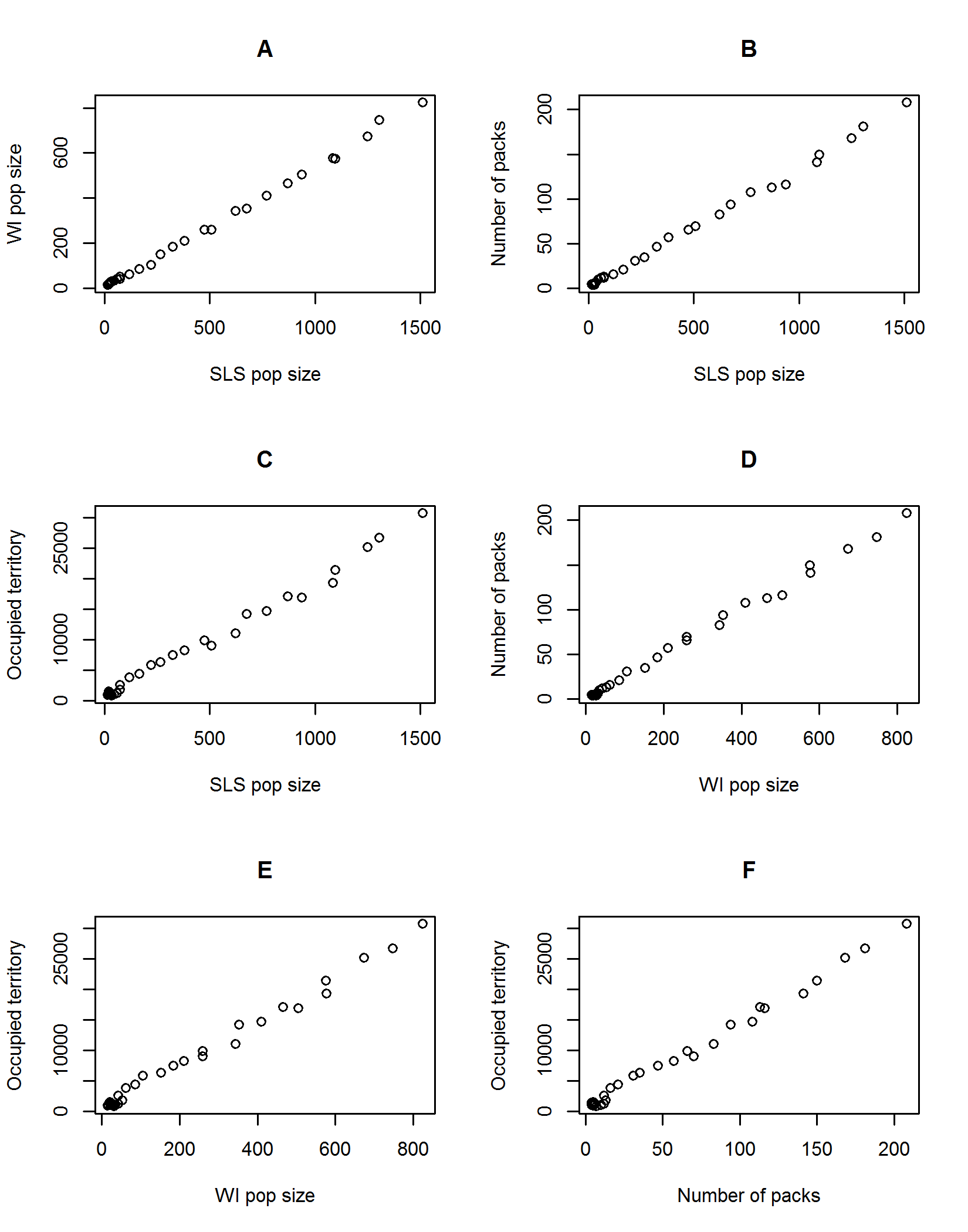


**Figure S1.1.** Correlation among 4 datasets (Table S1.1) of wolf population size in the southern Lake Superior (SLS) region, USA from 1980 – 2011.


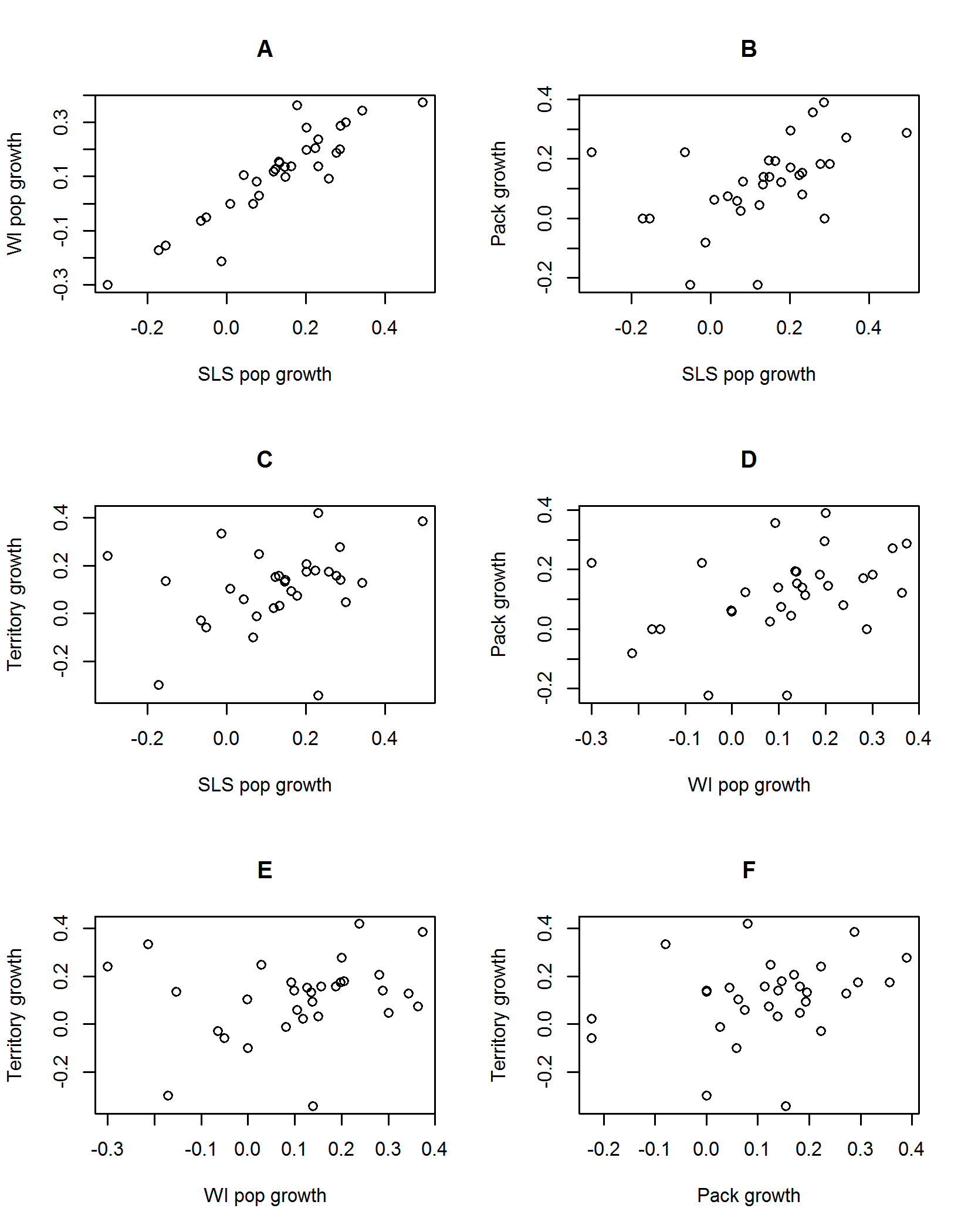


**Figure S1.2.** Correlation among 4 measures of wolf population growth calculated from 4 datasets of wolf population size (Table S1.1) in the southern Lake Superior (SLS) region, USA from 1980 – 2011.

**References**

1. Wydeven AP, Wiedenhoeft JE, Schultz RN, Thiel RP, Jurewicz RL, Kohn BE, et al. History, population growth, and management of wolves in Wisconsin. In: Wydeven AP, Van Deelen TR, Heske EJ, editors. Recovery of Gray Wolves in the Great Lakes Region of the United States: an endangered species success story. New York, New York, USA: Springer; 2009. p. 87-105.

2. Beyer DE, Jr., Peterson RO, Vucetich JA, Hammill JH. Wolf population changes in Michigan. In: Wydeven AP, Van Deelen TR, Heske EJ, editors. Recovery of Gray Wolves in the Great Lakes Region of the United States: An Endangered Species Success Story: Springer; 2009. p. 65-85.

3. MacFarland D, Wiedenhoeft JE. Wisconsin gray wolf post-delisting monitoring: 27 January 2012 through 14 April 2013. Madison, Wisconsin: Bureau of Wildlife Management, Wisconsin Department of Natural Resources; 2013.
