# Supplementary material for: Demographic and Component Allee Effects in Southern Lake Superior Gray Wolves
Source: PLoS One. 2016 Mar 1;11(3):e0150535. doi: 10.1371/journal.pone.0150535 (PMC4801012; doi:10.1371/journal.pone.0150535)
Supplement: S2 Appendix — (DOCX) [file pone.0150535.s002.docx]

**S2 Appendix.** Spline model with R code.

We used a low-rank thin-plate spline to model the relationship between ${pgr}_{i,t}$ and $ln(N_{t})$ for dataset $i$ where $i=1, 2, 3, 4$ (S1 Appendix) and year $t$ where $t=1981, 1982, \ldots, 2011.$ [Crainiceanu, Ruppert (1)](#_ENREF_1) provides background, implementation, and R and WinBUGS code for Bayesian analysis of penalized spline regression. We modified their code for our model, and refer you [Crainiceanu, Ruppert (1)](#_ENREF_1) for additional details about this approach.

Penalized spline regression allowed us to implement a nonparametric approach instead of a parametric approach to solving our problem because the shape of the curve on the graph of per capita growth rate versus population size (or some other measure) from an Allee effect does not necessarily take a predictable form. Fitting a penalized spline to the different measures of per capita growth versus population size did not require us to assume the shape of the Allee effect graph. We could have assumed the Allee effect graph was linear and fit a line to assess whether the slope of the line was positive. Alternatively, we could have assumed the Allee effect graph was parabolic and concave in shape and fit a quadratic function which would require perfect symmetry. There are other parametric functions that we could have fit, but this approach seemed to just answer the question of which parametric function we tried would fit the best. This did not necessarily translate into whether our ‘best fit’ parametric function was a good fit to the data. Instead, we took a non-parametric approach and did not assume that we knew the shape of the data. We used a penalized spline to fit a smooth function to the data and assessed whether that function demonstrated evidence for a demographic Allee effect.

We chose a Bayesian framework because it is straightforward and convenient to implement penalized splines and fit nonparametric regression models [[1](#_ENREF_1), [2](#_ENREF_2)]. Additionally, estimates of error are easily derived from the differences in posterior estimates at each iteration in the MCMC chains [[3](#_ENREF_3)]. We provide the model and R code with the data for southern Lake Superior (SLS) wolf population growth from 1980 – 2011. The other data can be found in Table S1.1 in S1 Appendix and can be substituted into the code to produce the other 3 models. Prior to running the following code, you will need to install JAGS version 3.3.0 and program R version 2.14 or later. Also, you will need to install library ‘rjags’ and library ‘coda’ in program R.

## Load libraries and navigate to working directory

library(rjags)

library(coda)

setwd(file.choose())

## Save the model into the working directory

sink("model.txt") # Saves the model into the directory

cat("

model {

for (t in 1:years) {

pgr[t] ~ dnorm(mu[t],tau)

mu[t] <- mfe[t] + mre[t]

mfe[t] <- beta1 * covariate[t]

mre[t] <-b[1]*Z[t,1]+b[2]*Z[t,2]+b[3]*Z[t,3]+b[4]*Z[t,4]+b[5]*Z[t,5]+b[6]*Z[t,6]+b[7]*Z[t,7]+b[8]*Z[t,8]+b[9]*Z[t,9]+b[10]*Z[t,10]+b[11]*Z[t,11]+b[12]*Z[t,12]+b[13]*Z[t,13]+b[14]*Z[t,14]+b[15]*Z[t,15]+b[16]*Z[t,16]+b[17]*Z[t,17]+b[18]*Z[t,18]+b[19]*Z[t,19]+b[20]*Z[t,20]

}

beta1 ~ dnorm(0,0.0001)

tau <- 1/(sd*sd)

sd ~ dunif(0,100)

taub <- 1/(sdb*sdb)

sdb ~ dunif(0,100)

for (i in 1:20) {

b[i]~dnorm(0,taub)

}

for (i in 1:T) {

mu2[i] <- mfe2[i] + mre2[i]

mfe2[i] <- beta1 * X3[i,2]

mre2[i] <- b[1]*Z3[i,1]+b[2]*Z3[i,2]+b[3]*Z3[i,3]+b[4]*Z3[i,4] + b[5]*Z3[i,5] + b[6]*Z3[i,6] + b[7]*Z3[i,7] + b[8]*Z3[i,8] + b[9]*Z3[i,9] + b[10]*Z3[i,10] + b[11]*Z3[i,11] + b[12]*Z3[i,12] + b[13]*Z3[i,13] + b[14]*Z3[i,14] + b[15]*Z3[i,15] + b[16]*Z3[i,16] + b[17]*Z3[i,17] + b[18]*Z3[i,18] + b[19]*Z3[i,19] + b[20]*Z3[i,20]

}

}

", fill = TRUE)

sink()

## Prepare the dataset

pop <- c(28,24,27,20,19,16,15,20,27,34,44,58,73,72,118,166,221,264,323,380,475,508,621,674,770,870,938,1086,1096,1250,1304,1511)

N0 = pop[1:31]

N1 = pop[2:32]

pgr = log(N1/N0)

# Create Zs for spline portion of the model

value = pgr

covariate = log(N1)

num.knots = 20

power = 3

N = length(covariate)

X <- cbind(rep(1,N),covariate,value)

knots<-quantile(unique(covariate),seq(0,1,length=(num.knots+2))[-c(1,(num.knots+2))])

Z_K<-(abs(outer(covariate,knots,"-")))^power

OMEGA_all<-(abs(outer(knots,knots,"-")))^power

svd.OMEGA_all<-svd(OMEGA_all)

sqrt.OMEGA_all<-t(svd.OMEGA_all$v %*%(t(svd.OMEGA_all$u)*sqrt(svd.OMEGA_all$d)))

Z<-t(solve(sqrt.OMEGA_all,t(Z_K)))

Z <- Z - mean(Z)

# Create new dataset for predicted $\mu$s to plot

newdate = seq(min(covariate),max(covariate)+.1,0.1)

Z3_K<-(abs(outer(newdate,knots,"-")))^power

Z3<-t(solve(sqrt.OMEGA_all,t(Z3_K)))

Z3 <- Z3 - mean(Z3)

T = length(newdate)

X3<-cbind(rep(1,T),newdate)

## Run the model

model = "model.spline.txt"

data = list(Z = Z, Z3 = Z3, X3 = X3, T = T,covariate = covariate, years=31,pgr=pgr)

params = c("beta1","mu2","sd","sdb")

jags = jags.model(model,data,n.chains=3,n.adapt=100000)

out = coda.samples(jags,params,50000)

summary(out)

**References**

1. Crainiceanu CM, Ruppert D, Wand MP. Bayesian Analysis for Penalized Spline Regression Using WinBUGS. Journal of Statistical Software. 2005;14:14.

2. Ruppert D, Wand MP, Carroll RJ. Semiparametric regression: Cambridge University Press; 2003.

3. Gelman A, Carlin JB, Stern HS, Rubin DB. Bayesian data analysis: CRC press; 2003.
