# Supplementary material for: Correction: Demographic and Component Allee Effects in Southern Lake Superior Gray Wolves
Source: PLoS One. 2022 May 26;17(5):e0269290. doi: 10.1371/journal.pone.0269290 (PMC9135252; doi:10.1371/journal.pone.0269290)
Supplement: S3 Appendix — (DOCX) [file pone.0269290.s001.docx]

**S3 Appendix.** Analysis of changes in fecundity and proportion of lone wolves over time.

We assessed three variables that could explain a reduction in early establishment of the Wisconsin, USA wolf population using data from Table 6.2 and 6.3 of Wydeven et al. 2009 (reproduced here as Table S3.2):

1. Reduced fecundity resulting in fewer pups per pack pre-1995 compared to 1995-2007
2. Reduced fecundity due a lower proportion of breeding females in the population pre-1995 compared to 1995-2007
3. Higher proportion of lone wolves pre-1995 compared to 1995-2007

For each variable of interest, we calculated a t-test to determine whether there was a difference in the mean of the variable for the years 1980-1994 compared to the mean of the variable from 1995-2007 (Table S3.1). There were no differences in means for fecundity measures. There was a significantly higher percentage of loner wolves pre-1995 compared to 1995-2007 whether the first two years of data (1980-1981) were treated as zeros or missing data (Table S3.1).

**Table S3.1.** Results of t-tests testing for differences of variables of fecundity and proportion of lone wolves in the Wisconsin, USA wolf population, 1980-2007.

| Variable | Mean 1980-1994 | Mean 1995-2007 | t-test statistic | p-value |
| --- | --- | --- | --- | --- |
| Pups per pack | 1.3 | 1.3 | -0.4 | 0.7 |
| Proportion breeding females | 0.2 | 0.3 | -0.8 | 0.5 |
| Percent lone wolves, with 1980 and 1981 as zeros | 9.0 | 3.8 | 4.0 | <0.001 |
| Percent lone wolves, with 1980 and 1981 as NA | 10.4 | 3.8 | 6.7 | <0.001 |

**Table S3.2.** Data on fecundity and the proportion of lone wolves over time for Wisconsin, USA. Data are reproduced from Tables 6.2 and 6.3 of Wydeven et al. 2009.

| Year | Max pop est^1^ | Packs^1^ | Prop breeding females^2^ | Midpoint pup est^3^ | Pups per pack^4^ | Loners (%)^5^ |
| --- | --- | --- | --- | --- | --- | --- |
| 1980 | 28 | 5 | 0.18 | 12 | 2.4 | 0? |
| 1981 | 24 | 5 | 0.21 | 7 | 1.4 | 0? |
| 1982 | 27 | 4 | 0.15 | 9 | 2.3 | 9 |
| 1983 | 20 | 5 | 0.25 | 5 | 1.0 | 11 |
| 1984 | 19 | 4 | 0.21 | 6 | 1.5 | 11 |
| 1985 | 16 | 4 | 0.25 | 4 | 1.0 | 7 |
| 1986 | 15 | 5 | 0.33 | 3 | 0.6 | 13 |
| 1987 | 20 | 5 | 0.25 | 6 | 1.2 | 11 |
| 1988 | 27 | 6 | 0.22 | 9 | 1.5 | 12 |
| 1989 | 31 | 7 | 0.23 | 11 | 1.6 | 10 |
| 1990 | 34 | 10 | 0.29 | 8 | 0.8 | 9 |
| 1991 | 41 | 12 | 0.29 | 13 | 1.1 | 5 |
| 1992 | 52 | 13 | 0.25 | 13 | 1.0 | 13 |
| 1993 | 42 | 12 | 0.29 | 10 | 0.8 | 15 |
| 1994 | 61 | 16 | 0.26 | 16 | 1.0 | 9 |
| 1995 | 86 | 21 | 0.24 | 26 | 1.2 | 11 |
| 1996 | 105 | 31 | 0.30 | 31 | 1.0 | 3 |
| 1997 | 151 | 35 | 0.23 | 61 | 1.7 | 3 |
| 1998 | 184 | 47 | 0.26 | 66 | 1.4 | 3 |
| 1999 | 211 | 57 | 0.27 | 68 | 1.2 | 3 |
| 2000 | 259 | 66 | 0.25 | 88 | 1.3 | 5 |
| 2001 | 259 | 70 | 0.27 | 88 | 1.3 | 3 |
| 2002 | 343 | 83 | 0.24 | 120 | 1.4 | 2 |
| 2003 | 353 | 94 | 0.27 | 110 | 1.2 | 4 |
| 2004 | 410 | 108 | 0.26 | 128 | 1.2 | 4 |
| 2005 | 465 | 113 | 0.24 | 155 | 1.4 | 3 |
| 2006 | 504 | 116 | 0.23 | 186 | 1.6 | 3 |
| 2007 | 577 | 138 | 0.24 | 190 | 1.4 | 3 |

^1^ Data from Table 6.2 of Wydeven et al. 2009. Packs are also interpreted as the number of breeding females in the population under the assumption that there is a single breeding female per pack.

^2^ Calculated column of packs (interpreted as proportion of breeding females) divided by upper end of the population estimate.

^3^ Data from Table 6.3 of Wydeven et al. 2009.

^4^ Calculated column of the pup estimates divided by packs.

^5^ Data from Table 6.2 of Wydeven et al. 2009.

**References**

1. Wydeven AP, Wiedenhoeft JE, Schultz RN, Thiel RP, Jurewicz RL, Kohn BE, et al. History, population growth, and management of wolves in Wisconsin. In: Wydeven AP, Van Deelen TR, Heske EJ, editors. Recovery of Gray Wolves in the Great Lakes Region of the United States: an endangered species success story. New York, New York, USA: Springer; 2009. p. 87-105.
